# Supplementary material for: Illusory resizing of the painful knee is analgesic in symptomatic knee osteoarthritis
Source: PeerJ. 2018 Jul 17;6:e5206. doi: 10.7717/peerj.5206 (PMC6054060; doi:10.7717/peerj.5206)
Supplement: File S1 [file peerj-06-5206-s002.docx]

**Considering the effect of first-person vs third-person perspective on Pain scores**

**1.** **Effect of VT illusion versus touch-only and vision-only components on pain:**

Condition (VT congruent illusion vs TO control vs VO control) x Time (pre-condition vs post-condition) x Perspective (first- vs third-person) Repeated Measures Analysis of Variance (RM ANOVA)

| **Factor** | **F-value** | **p-value** | **Partial Eta Squared** |
| --- | --- | --- | --- |
| Condition | F_2,20_ = 0.57 | 0.57 | 0.054 |
| Condition x Perspective | F_2,20_ = 0.449 | 0.64 | 0.043 |
| Time | F_1,10_ = 5.11 | **0.047*** | 0.39 |
| Time x Perspective | F_1,10_ = 1.41 | 0.26 | 0.12 |
| Condition x Time | F_2,20_ = 3.73 | **0.043*** | 0.27 |
| Condition x Time x Perspective | F_2,20_ = 0.455 | 0.64 | 0.044 |

While the interaction was not significant, below are the Pain scores for the Condition x Time x Perspective:

| **Condition** | **Time** | **Mean** | **Std Error** | **95% Confidence interval** |
| --- | --- | --- | --- | --- |
| **First-person perspective** | | | | |
| VT illusion | Pre | 28.3 | 8.4 | 9.5 – 47.1 |
|  | Post | 23.7 | 7.5 | 6.9 – 40.4 |
| TO condition | Pre | 25.8 | 8.2 | 7.6 – 44.0 |
|  | Post | 23.8 | 7.8 | 6.4 – 41.3 |
| VO condition | Pre | 22.7 | 8.9 | 2.7 – 42.6 |
|  | Post | 24.7 | 8.2 | 6.4 – 42.9 |
| **Third-person perspective** | | | | |
| VT illusion | Pre | 32.5 | 8.4 | 13.7 – 51.3 |
|  | Post | 21.7 | 7.5 | 4.9 – 38.4 |
| TO condition | Pre | 33.3 | 8.2 | 15.1 – 51.5 |
|  | Post | 30.8 | 7.9 | 13.3 – 48.3 |
| VO condition | Pre | 26.7 | 8.9 | 6.8 – 46.6 |
|  | Post | 25.0 | 8.2 | 6.7 – 43.3 |

|  |
| --- |

**2.** **Effect of congruent VT illusion versus Incongruent condition that controls for visual input:**

Condition (VT congruent illusion vs VT incongruent illusion [identical visual input, differing tactile input]) x Time (pre- vs post-condition) x Perspective (first- vs third-person) RM ANOVA.

| **Factor** | **F-value** | **p-value** | **Partial Eta Squared** |
| --- | --- | --- | --- |
| Condition | F_1,10_ = 0.033 | 0.86 | 0.03 |
| Condition x Perspective | F_1,10_ = 1.38 | 0.27 | 0.12 |
| Time | F_1,10_ = 11.45 | **0.007*** | 0.53 |
| Time x Perspective | F_1,10_ = 0.00 | 1.00 | 0.00 |
| Condition x Time | F_1,10_ = 0.41 | 0.54 | 0.039 |
| Condition x Time x Perspective | F_1,10_ = 3.28 | 0.10 | 0.24 |

While the interaction was not significant, below are the Pain scores for the Condition x Time x Perspective:

| **Condition** | **Time** | **Mean** | **Std Error** | **95% Confidence interval** |
| --- | --- | --- | --- | --- |
| **First-person perspective** | | | | |
| VT congruent illusion | Pre | 28.3 | 8.4 | 9.5 – 47.1 |
|  | Post | 23.7 | 7.5 | 6.9 – 40.4 |
| VT incongruent | Pre | 28.5 | 8.4 | 9.8 – 47.2 |
|  | Post | 19.8 | 8.4 | 1.2 – 38.5 |
| **Third-person perspective** | | | | |
| VT congruent illusion | Pre | 32.5 | 8.4 | 13.7 – 51.3 |
|  | Post | 21.7 | 7.5 | 4.9 – 38.4 |
| VT incongruent | Pre | 33.3 | 8.4 | 12.2 – 49.5 |
|  | Post | 28.3 | 8.4 | 9.7 – 47.0 |

**3.** **Effect of congruent VT illusion versus Incongruent condition that controls for touch input:**

Condition (VT congruent illusion vs VT incongruent illusion [identical tactile input, differing visual input]) x Time (pre- vs post-condition) x Perspective (first- vs third-person) RM ANOVA.

| **Factor** | **F-value** | **p-value** | **Partial Eta Squared** |
| --- | --- | --- | --- |
| Condition | F_1,10_ = 0.66 | 0.43 | 0.062 |
| Condition x Perspective | F_1,10_ = 0.061 | 0.81 | 0.006 |
| Time | F_1,10_ = 5.45 | **0.042*** | 0.35 |
| Time x Perspective | F_1,10_ = 1.45 | 0.26 | 0.13 |
| Condition x Time | F_1,10_ = 4.84 | 0.052 | 0.33 |
| Condition x Time x Perspective | F_1,10_ = 0.06 | 0.81 | 0.006 |

While the interaction was not significant, below are the Pain scores for the Condition x Time x Perspective:

| **Condition** | **Time** | **Mean** | **Std Error** | **95% Confidence interval** |
| --- | --- | --- | --- | --- |
| **First-person perspective** | | | | |
| VT congruent illusion | Pre | 28.3 | 8.4 | 9.5 – 47.1 |
|  | Post | 23.7 | 7.5 | 6.9 – 40.4 |
| VT incongruent | Pre | 22.7 | 7.3 | 5.3 – 38.0 |
|  | Post | 21.0 | 8.3 | 2.6 – 39.4 |
| **Third-person perspective** | | | | |
| VT congruent illusion | Pre | 32.5 | 8.4 | 13.7 – 51.3 |
|  | Post | 21.7 | 7.5 | 4.9 – 38.4 |
| VT incongruent | Pre | 27.5 | 7.3 | 11.2 – 43.8 |
|  | Post | 21.7 | 8.3 | 3.3 – 40.0 |
